# Primary central nervous system lymphoma presenting as multiple space-occupying lesions in advanced human immunodeficiency virus infection

**DOI:** 10.4102/sajr.v21i2.1234

**Published:** 2017-11-14

**Authors:** Sara Zafar, Maria Javed, Neesha Rockwood, Farhat Kazmi

**Affiliations:** 1 Radiology Department, Chelsea and Westminster Hospital, United Kingdom; 2 Department of HIV, Chelsea and Westminster Hospital, United Kingdom

## Abstract

A 31-year-old man presented with seizures and cerebellar symptoms on a background of weight loss and lethargy. He was found to be infected with human immunodeficiency virus (HIV) and following radiological imaging, was commenced on treatment for presumed cerebral toxoplasmosis. Due to a lack of response, both clinically and on interval imaging, a positron-emission tomography-computed tomography and brain biopsy were undertaken, which demonstrated high-grade primary central nervous system lymphoma (PCNSL). Awareness amongst both clinicians and radiologists of the multifarious patterns of intra-cranial involvement in patients with HIV is, therefore, of utmost importance, as the treatment and prognosis of these entities are entirely different.

## Introduction

Progressive immunosuppression secondary to human immunodeficiency virus (HIV) infection may predispose to opportunistic infections affecting the central nervous system (CNS). Patients may present with space-occupying lesions, such as toxoplasmosis, cryptococcal disease and tuberculosis, in addition to being associated with an increased incidence of Epstein–Barr virus (EBV) associated systemic or primary cerebral nervous system lymphoma (PCNSL). Distinguishing between these entities by an accurate interpretation of imaging along with the clinical presentation is therefore essential, as the course of treatment in these cases is entirely different.^1^

## Case report

A 31-year-old man presented to the emergency department in December 2016 with new-onset tonic–clonic seizures, on a background of 19 kg weight loss, progressive lethargy and cognitive decline over the preceding 18 months. He had travelled extensively across the African subcontinent whilst working aboard a ship and during this time had engaged in unprotected intercourse with female sex workers.

**FIGURE 1:**
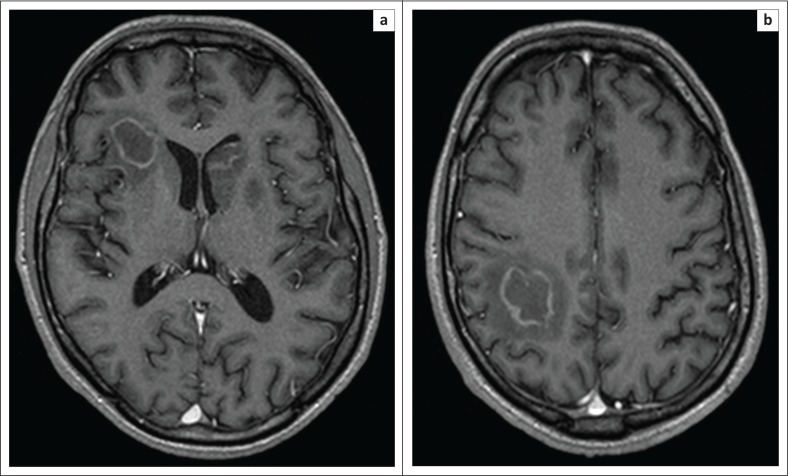
Post-gadolinium T1 contrast enhanced axial slices from MRI brain performed 2 days following the initial clinical presentation. Multiple ring-enhancing lesions are seen within the right frontal (a), left basal ganglia and corticomedullary junction within the right parietal lobe (b). Effacement of anterior horn of the left lateral ventricle is noted (a).

**FIGURE 2:**
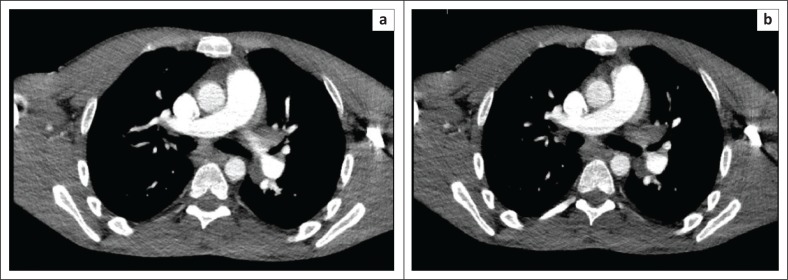
Axial slices from portal venous phase contrast enhanced CT thorax, abdomen and pelvis performed at initial presentation, which demonstrates mediastinal (a) and hilar (b) lymph node enlargement. These nodes were later not found to be FDG-avid on FDG-PET-CT.

Salient examination findings included nystagmus on right-lateral gaze, past-pointing in the left upper limb, brisk reflexes and an unsteady gait, suggestive of underlying cerebellar pathology. Following biochemical and serological investigations, the patient was diagnosed with HIV-1, with a viral load of 1 243 938 copies/mL and a CD4 lymphocyte count of 12 cells/µL. Initial computed tomography (CT) imaging revealed multiple lesions in the brainstem, cerebellum, basal ganglia and both cerebral hemispheres, with significant local mass effect and effacement of the left lateral ventricle. Magnetic resonance imaging (MRI) of the brain was also undertaken at this time, which demonstrated multiple ring-enhancing lesions (Figure 1). Mediastinal and hilar lymph node enlargement identified on CT imaging of the chest, abdomen and pelvis was felt to be in keeping with persistent generalised lymphadenopathy (PGL) because of advanced HIV as there was no palpable cervical, axillary or inguinal lymphadenopathy (Figure 2).

The patient was commenced on treatment for presumptive toxoplasmosis, with sulfadiazine/pyramethamine/folinic acid and adjunctive high-dose dexamethasone, levetiracetam. He also simultaneously commenced antiretroviral therapy (ART) comprising of tenofovir/emtricitabine and dolutegravir, along with prophylactic antibiotics and antifungals against opportunistic infections. Blood cultures were negative as was toxoplasma serum Immunoglobulin G (IgG). Tuberculosis with CNS involvement and cryptococcoma were considered as differential diagnoses; however, cryptococcal antigen was negative as subsequently were mycobacterial blood and induced sputum cultures. Cerebrospinal fluid examination was not attempted in light of the cerebellar lesions.

**FIGURE 3:**
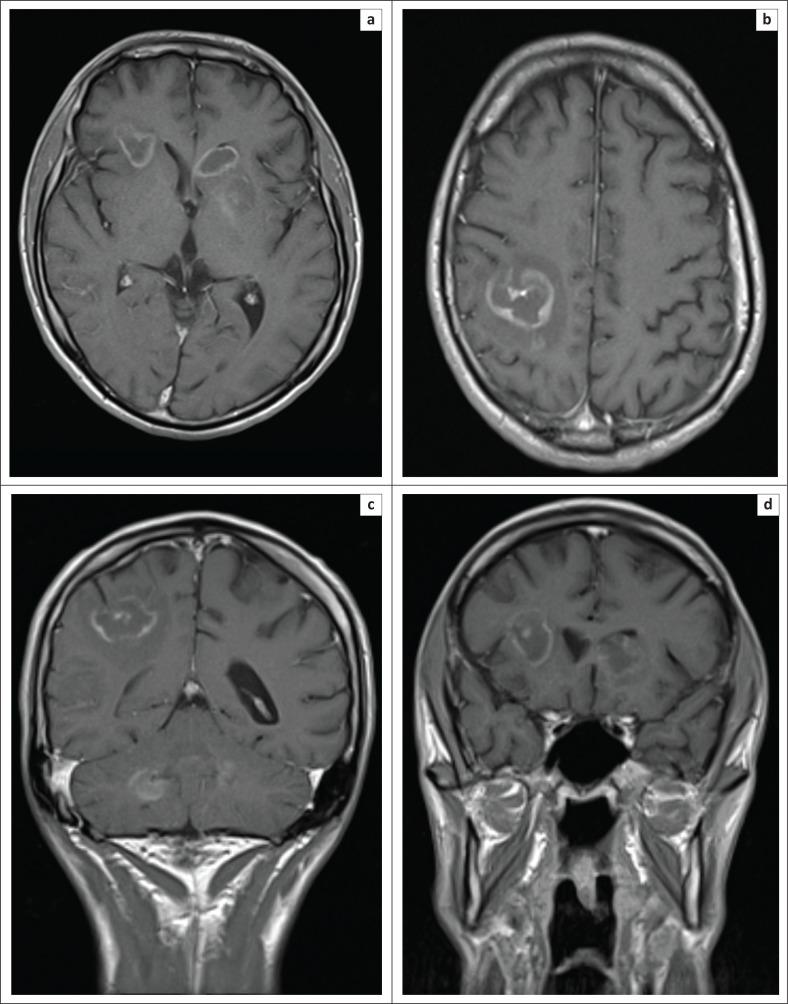
Axial (a, b) and coronal (c, d) slices from T1 post-contrast enhanced MR brain 2 weeks following the initial imaging. No significant treatment response following 2 weeks of toxoplasmosis treatment with sulfadiazine/pyramethamine/folinic acid and adjunctive high-dose dexamethasone with persistent ring-enhancing lesions identified. There was marked peri-lesional vasogenic oedema and two new lesions were seen. A few of these lesions, for example, the lesion within the head of the left caudate nucleus demonstrated restricted diffusion on diffusion weighted imaging (not demonstrated), in keeping with increased cellularity. This finding is atypical of toxoplasmosis and thus raised the suspicion for alternative diagnoses over toxoplasma.

Following 2 weeks of toxoplasmosis treatment, there was no improvement in the appearances of the intra-cranial lesions on repeat MR brain imaging, with two new lesions being identified (Figure 3). At this point, alternative diagnoses such as systemic lymphoma with intra-cranial metastases and PCNSL were pursued over toxoplasmosis. A fluodeoxyglucose positron-emission tomography (FDG-PET-CT) and brain biopsy were, thus, arranged with the aim of obtaining a more definitive diagnosis.

FDG-PET-CT was undertaken 3 weeks following initial presentation and demonstrated high avidity activity only in the brain including the right parietal, right parieto-occipital and left frontal regions as well as the cerebellum and basal ganglia (standardised uptake value [SUV] max 7.3 compared with normal brain cortex). No FDG-avid mediastinal lymph nodes were identified and these appearances pointed towards the diagnosis of PCNSL, rather than systemic lymphoma with cerebral metastases.

The right parietal lobe brain biopsy demonstrated a suspicious lymphoid infiltrate. Mycobacterial and fungal stains were negative as was immunostaining for herpes simplex virus (HSV1), HSV2, cytomegalovirus (CMV) and John Cunningham virus (JCV). Epstein–Barr encoding region (EBER) *in situ* hybridisation staining was also negative; however, this was a likely false negative result, in the context of necrosis and ribonucleic acid (RNA) degradation. Within the necrotic areas, the cellular outlines and remains were strongly positive for CD20, CD79a and BCL2. They were negative for CD19, CD3, CD5, CD10, BCL6 and PAX5. Final histopathology results were in keeping with a high-grade B-cell lymphoma that was necrotic.

**FIGURE 4:**
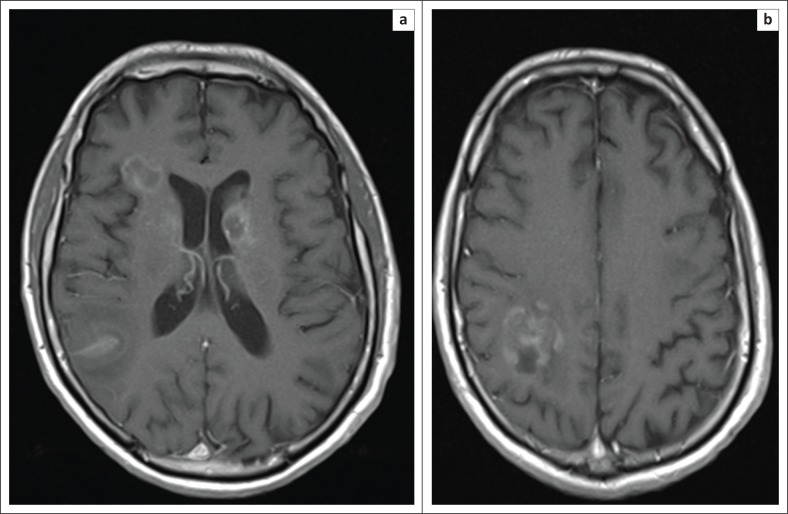
Post-gadolinium contrast enhanced T1 axial slices from MR brain performed 2 months following the initial clinical presentation. The patient had been commenced on MATRix chemotherapy. Multiple peripherally enhancing parenchymal (b) and sub-ependymal lesions (a) are demonstrated with a significant improvement in the size of all lesions since the last examination. New high signal on T1 imaging (not demonstrated) may represent post-treatment interval haemorrhage.

The patient was commenced on MATRix chemotherapy (rituximab, methotrexate, cytarabine, with omission of thioTEPA for the first two cycles) for HIV-associated primary cerebral lymphoma.^2^ Within 8 weeks of commencement of highly active antiretroviral therapy (HAART), there was a 3log10 fold decrease in HIV-1 viral load. There has also been a marked improvement in cognitive function and gait following four cycles of MATRix chemotherapy (Figure 4).

**FIGURE 5:**
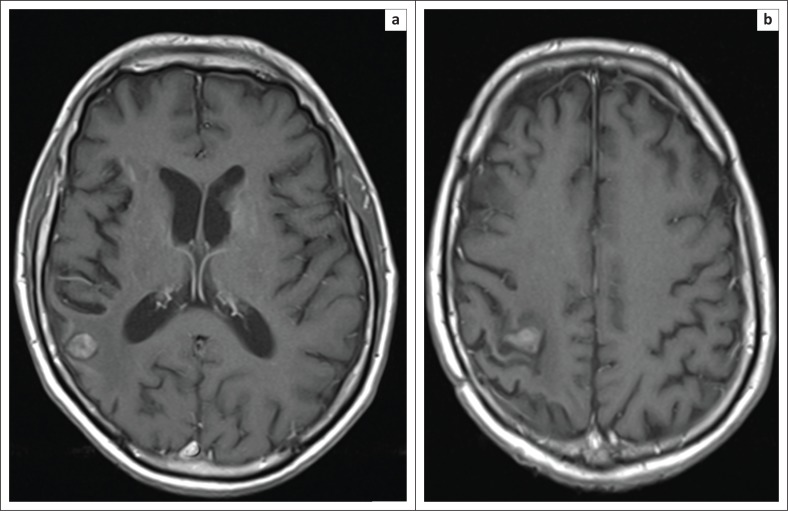
Post-gadolinium contrast enhanced T1 axial slices from MR brain following four cycles of MATRix chemotherapy, which shows marked interval improvement in both the size of the ring-enhancing lesions (a) and the degree of peri-lesional oedema (b), in keeping with positive disease response.

Repeat MRI imaging 6 months following initial presentation also demonstrates a reduction in volume of the supra and infra-tentorial parenchymal and sub-ependymal lesions, with improvement in the degree of peri-lesional vasogenic oedema, in keeping with a positive disease response (Figure 5). The patient continues to make good progress and is currently being worked up for consolidative therapy with an autologous stem cell transplantation.

## Discussion

Toxoplasmosis and PCNSL are amongst the most common causes of intra-cranial space-occupying lesions in patients with HIV infection.^1^ Distinguishing between these two aetiologies may sometimes be challenging because of a degree of overlap in the spectra of imaging findings. A constellation of imaging modalities including CT, MRI, perfusion MR, FDG-PET and Thallium-201 single photon emission computed tomography (SPECT), along with brain biopsy, can therefore be utilised collectively to afford the clinical team the best chance at making the correct diagnosis and initiate appropriate treatment in a timely manner.

There is a broad list of differential diagnoses for a ring-enhancing lesion in a patient with HIV including infection (toxoplasma, tuberculous abscesses, tuberculoma and cystercercosis), neoplasia (PCNSL, metastases), demyelination (MS) and vascular disease (haematoma). Whilst the presence of multiple ring-enhancing lesions are characteristically associated with toxoplasmosis, a solitary, homogenously-enhancing lesion more frequently typifies primary cerebral lymphoma. Occasionally, lymphomatous lesions may demonstrate areas of central necrosis with thicker peripherally enhancing portions, thereby masquerading as ring-enhancing lesions, giving rise to the difficulty distinguishing these two causes, as was the case with our patient.^3^

The distribution of lesions may be another helpful factor in aiding discrimination, with a preponderance of toxoplasma to the basal ganglia, thalami and cortico-medullary-junction. Lymphoma favours a peri-ventricular location with a classic sub-ependymal route of spread. Haemorrhage may occasionally be seen in necrotic toxoplasmosis lesions, however, this may also be identified as T1 hyperintensity in patients with lymphoma following the initiation of treatment, as was the case in our patient. (Figure 4)

Diffusion weighted imaging (DWI) also highlights specific differences between these two aetiologies: lymphomatous lesions demonstrate marked restricted diffusion because of the hypercellularity of these lesions, whilst the converse is true of toxoplasma.^4^ The intra-cranial lesions in our case demonstrated hyperintensity on the DWI, with corresponding central hypointensity on the apparent diffusion coefficient (ADC) map, in keeping with a diagnosis of lymphoma rather than toxoplasma.

Nuclear medicine imaging offers further clues in differentiation, as toxoplasma demonstrates decreased uptake on Thallium-201 SPECT imaging, whereas uptake is increased in lymphoma.^5^ FDG-PET can also be of value, with lymphomatous lesions demonstrating high metabolic activity and toxoplasmosis and other infections demonstrating hypometabolic features. This was particularly useful in our case.^6^

## Conclusion

A wide differential diagnosis exists for intra-cranial mass lesions in HIV patients. It can be challenging to distinguish toxoplasmosis from PCNSL, which are two common CNS lesions. Whilst MRI and nuclear medicine provide us with clues to facilitate identifying the underlying aetiology of an intra-cranial mass lesion, invasive brain biopsy may still be required because of the significant overlap in the spectra of imaging findings. Utilising nuclear medicine to aid diagnosis may also prevent delays in initiating focused treatment.
